# Monitoring the Phenolic Ripening of Red Grapes Using a Multisensor System Based on Metal-Oxide Nanoparticles

**DOI:** 10.3389/fchem.2018.00131

**Published:** 2018-04-24

**Authors:** Celia Garcia-Hernandez, Cristina Medina-Plaza, Cristina Garcia-Cabezon, Yolanda Blanco, Jose A. Fernandez-Escudero, Enrique Barajas-Tola, Miguel A. Rodriguez-Perez, Fernando Martin-Pedrosa, Maria L. Rodriguez-Mendez

**Affiliations:** ^1^Group UVaSens, Department of Inorganic Chemistry, Escuela de Ingenierías Industriales, Universidad de Valladolid, Valladolid, Spain; ^2^Group UVasens, Department of Materials Science, Universidad de Valladolid, Valladolid, Spain; ^3^Estacion Enologica de Castilla y Leon, Valladolid, Spain; ^4^ITACYL, Valladolid, Spain

**Keywords:** electronic tongue, grape, ripening, phenolic maturity, metal oxide nanoparticles

## Abstract

The maturity of grapes is usually monitored by means of the sugar concentration. However, the assessment of other parameters such as the phenolic content is also important because the phenolic maturity has an important impact on the organoleptic characteristics of wines. In this work, voltammetric sensors able to detect phenols in red grapes have been developed. They are based on metal oxide nanoparticles (CeO_2_, NiO, and TiO_2_,) whose excellent electrocatalytic properties toward phenols allows obtaining sensors with detection limits in the range of 10^−8^ M and coefficients of variation lower than 7%. An electronic tongue constructed using a combination of the nanoparticle-based sensors is capable to monitor the phenolic maturity of red grapes from véraison to maturity. Principal Component Analysis (PCA) can be successfully used to discriminate samples according to the ripeness. Regression models performed using Partial Least Squares (PLS-1) have established good correlations between voltammetric data obtained with the electrochemical sensors and the Total Polyphenolic Index, the Brix degree and the Total Acidity, with correlation coefficients close to 1 and low number of latent variables. An advantage of this system is that the electronic tongue can be used for the simultaneous assessment of these three parameters which are the main factors used to monitor the maturity of grapes. Thus the electronic tongue based on metal oxide nanoparticles can be a valuable tool to monitor ripeness. These results demonstrate the exciting possible applications of metal oxide nanoparticles in the field of electronic tongues.

## Introduction

Grapes must be harvested at the optimal maturity point. The sugar content has a direct influence on the alcoholic degree of wines and it is the analytical indicator commonly used to decide the harvest date (OIV, 2013). It is periodically assessed using fast and simple density measurements and it is expressed as degree Brix (°Brix). The variation of the acidity along ripening is also an important parameter which is expressed as Total Acidity (TA) (Boss et al., 2014).

The phenolic content of grapes also changes during ripening and the phenolic maturity has a direct impact on the organoleptic characteristics of wines, and it would be desirable to assess it routinely. However, the main changes in the phenolic content take place in skins and seeds, and the assessment of phenolic maturity requires the previous separation of seeds and skins using long and complex methods (Xu et al., 2011; Meléndez et al., 2013; Nogales-Bueno et al., 2014; Sharma et al., 2015). Oenologists require new methods capable to measure the phenolic maturity of grapes with simple and direct methods.

Besides, it would be of great interest to develop new technologies able to detect simultaneously the sugar content, acidity and phenolic composition. Such a method would help to take faster decisions about the optimal harvest date. One possible approach can be the use of holistic methods, where complex signals obtained from an instrument such as GC-MS, FTIR or NMR, are processed using multivariate methods. These holistic methods have been successfully used to classify wines according to quality, to monitor fermentation, to detect wine spoilage, etc. (Buratti et al., 2011; Cozzolino et al., 2012; Godelmann et al., 2014).

Electronic tongues (ET) are a new class of instruments which are gaining interest in the food industry (Cosio et al., 2012; Kirsanov et al., 2012; Lvova et al., 2014; Rodríguez-Méndez, 2016). They are multisensor systems where an array of sensors is combined with a multivariate data software. The most common sensors used in ETs dedicated to the analysis of wines are electrochemical (amperometric or voltammetric). They have been widely used to discriminate wines according to the variety of grape, the vintage, the type of barrel used for aging etc. (Gutiérrez et al., 2010; Prieto et al., 2011; Apetrei et al., 2012; Cetó et al., 2017; Rudnitskaya et al., 2017). In spite of the interest of winemakers to have available objective methods to determine the harvesting date and the quality of grapes, ETs have been rarely applied to the quality control of grapes or their musts (Codinachs et al., 2008; Gutiérrez et al., 2011; Campos et al., 2013; Medina-Plaza et al., 2014a,b).

Nowadays, it is clear that the performance of ETs can be improved by introducing in the array sensing units specifically dedicated to a particular application. Electrodes with enhanced performances can be obtained using new sensing materials linked to nanotechnology. Metal nanoparticles have been used in the fabrication of electrochemical sensors due to their electrocatalytic properties which are related to the formation of mixed valence states on their surface (Campbell and Compton, 2010; Saha et al., 2012; Lin et al., 2013). Metal nanoparticles have demonstrated their capability to catalyze the oxidation/reduction of organic acids and phenols commonly present in wines. The oxidation of phenols occurs at lower potentials, whereas the presence of metallic nanoparticles increases the reactivity of acids and enhances the intensity of the anodic wave at negative potentials (Medina-Plaza et al., 2015a,b). In spite of this interest, metal nanoparticles have rarely been included as sensing materials in electronic tongues (Gutiérrez et al., 2013; Sharpe et al., 2014; Mercante et al., 2015). Up to now, the sensing capabilities of metal oxide nanoparticles (MONPs) have never been tested in electronic tongues.

The aim of this work was to develop an electronic tongue (ET) formed by sensors based on metal oxide nanoparticles (CeO_2_, NiO, and TiO_2_,) and to evaluate their capabilities to detect phenols. Using chemometric techniques such as Principal Component Analysis (PCA), the ET was used to monitor the ripeness of 8 different varieties of grapes, from *véraison* (the onset of ripening) to post-maturity. Mathematical models were built to establish correlations between the phenolic index measured by traditional chemical techniques and the results of the ET. PLS-1 was used to predict the polyphenol index of the grapes along ripening. The possibility to obtain information about other classical indicators of maturity such as the °Brix and Total Acidity was also analyzed.

## Materials and methods

Metal oxide nanoparticles of Titanium (IV), Cerium (IV), and Nickel (II) (TiO_2_NP, CeO_2_NP, NiONP) were purchased from Sigma-Aldrich. All reagents and solvents were of high purity (Sigma–Aldrich). Deionized water (18.2 MΩ·cm^−1^) was obtained from a Millipore MilliQ purifier.

Electron Microscope TEM images were recorded using a high-resolution electron microscope (HRTEM: JEOL JEM 2200) (Tokyo, Japan) operating at an accelerating voltage of 200 kV. Sample images were processed using Image J image processing software (public software).

Eight varieties of red grapes typical from the Castilla-Leon region (Spain) were included in the study: *Tempranillo, Garnacha, Cabernet-Sauvignon, Prieto Picudo, Mencia Regadio, Mencia Secano, Rufete*, and *Juan Garcia*. Grapes were grown and harvested by the Agrotechnological Institute of Agriculture of the Regional Government (ITACyL) and the cellar Bodega Cooperativa de Cigales. Berries were collected in a weekly basis from *véraison* until grapes were completely mature. Ripeness rate was different from one variety to another. For this reason, the official harvest date indicated by traditional chemical parameters, varied from one variety of grape to another. Grapes from Tempranillo, Garnacha, and Cabernet Sauvignon were considered matured 6 weeks after *véraison*. The rest of varieties attained the optimal ripeness 5 weeks after *véraison*. In all cases several grape bunches were left in the vine and two extra samplings were collected in successive weeks in order to analyze over-ripened grapes.

Musts were prepared from 100 berries representative of the ripening state of the vineyard. Once collected, grapes were crushed, and peels and seeds were separated by decantation. 50 mL aliquots of musts thus obtained, were frozen at −20°C until used. Total Polyphenol Index (TPI), Brix degree (°Brix) and Total Acidity (TA) were analyzed following international standard methods (OIV, 2013; Boss et al., 2014). TPI was determined by diluting the samples with distilled water in a 1:100 ratio and measuring the absorbance directly at 280 nm (using a quartz cuvette of 10 mm optical path). The value of the I280 nm (TPI) was calculated by multiplying the absorbance × 100. The total acidity is the sum of its titratable acidities when it is titrated to pH 7 against a standard alkaline solution. Carbon dioxide is not included in the total acidity. The total acidity is usually expressed in grams of tartaric acid per liter. Brix measurements are taken with an Atago WM-7 digital refractometer.

**Figure 1:**
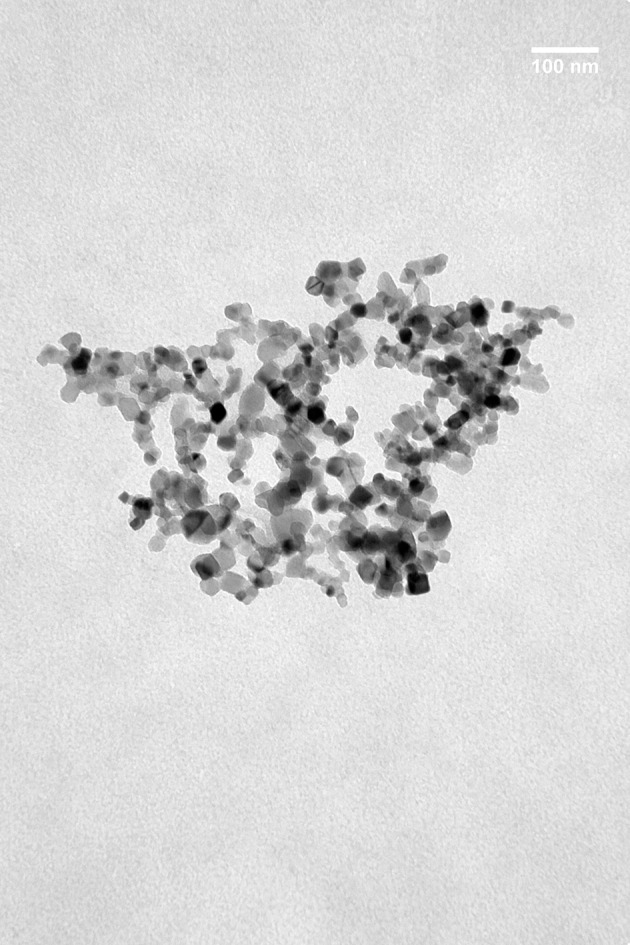
TEM image of the TiO_2_NPs.

A multisensor system consisting of 4 Carbon Paste Electrodes (CPE) was formed. Three electrodes were modified with metal oxide nanoparticles including NiO (NiONP-CPE), TiO_2_ (TiO_2_NP-CPE), and CeO_2_ (CeO_2_NP-CPE). One unmodified carbon paste electrode (C-CPE) was also included in the array. Electrodes were prepared following the classical method by mixing graphite with the corresponding metal oxide nanoparticle (15% w/w of the MONP) Nujol was used as the binder until a paste was obtained. The paste was packed in a 1 mL plastic syringe and compressed. A metallic copper wire was used as a contact (Apetrei et al., 2011). The size of MNOPS ranged from 20 to 50 nM. Figure 1 illustrates the shape and size of TiO_2_MNPs. As observed in the TEM image, MONPs were spheric with an homogeneous distribution of sizes that coincided with the size labeled by the supplier.

Electrochemical experiments were carried out using a potentiostat (Autolab. Metrohm, USA) combined with a multiplexor system. CPEs (surface of 2 mm in diameter) served as working electrodes, with an Ag|AgCl/KCl 3 mol·L^−1^ electrode acting as a reference electrode and a platinum wire as the counter electrode. The MONPs sensors were able to provide responses toward aqueous solutions of phenolic acids (vanillic and caffeic acids) and polyphenols (catechol and pyrogallol) present in wines. The electrocatalytic properties and the detection limits (LD) were evaluated. The calibration curves were constructed measuring standard solutions with concentrations ranging from 10^−4^ to 10^−6^ mol·L^−1^. LDs were calculated following the 3 *SD*/m criterion. The reproducibility of data provided by the MONPs sensors was evaluated by comparing data provided by three sensors measuring standard solutions in different days. Cyclic voltammograms were registered at a sweep rate of 0.1 V·s^−1^ from −0.8 to +1.0 V. Four replicas of each sample were measured. Principal Component Analysis (PCA) and Partial Least Squares (PLS) models were built using the software Matlab v5.3. (The Mathworks Inc., Natick, MA, USA).

Prior to perform the statistical analysis, the number of variables was reduced using a data reduction technique based on predefined response “bell-shaped-windowing” curves called “kernels” (Medina-Plaza et al., 2015b). In this method, each voltammogrammetric curve is multiplied by 10 smooth, bell-shaped windowing functions defined as:

$${\text{K}}_{\text{i}}({\text{V}}_{\text{j}})\;=\;\frac{1}{1+{\left(\frac{{\text{V}}_{\text{j}}-{\text{c}}_{\text{i}}}{{\text{a}}_{\text{i}}}\right)}^{{\text{2b}}_{\text{i}}}}$$

where a_i_, b_i_, and c_i_ define the width, shape, and center of the different windowing functions K_i_. Subsequently, data were integrated with respect to voltage. As a result, each voltammogram was reduced to a vector of 10 variables that were used as the input data source for statistical analysis.

## Results and discussion

### Monitoring ripeness using chemical parameters

**Figure 2:**
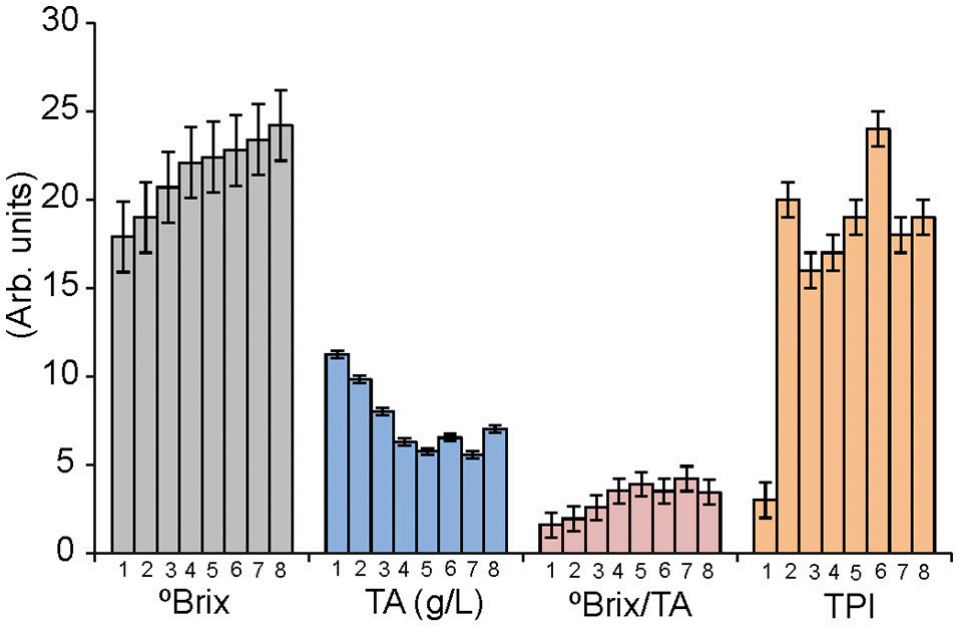
Variation of the °Brix (g sugar/ 100 g solution), TA (g/L), Brix/TA and TPI for juices obtained from the variety Tempranillo harvested weekly form *véraison* to over-ripening.

Ripening was monitored by measuring °Brix, TA, and TPI in a weekly basis (from *véraison*). These parameters were used by oenologists to establish the optimal date of harvest. The °Brix/TA ratio was also calculated. This parameter is also commonly used to decide the optimal maturity date even if there is no biochemical relationship between the acidity loss and the sugar increase. The complete list of parameters is collected in Supplementary Table. Details can be observed in Figure 2 for variety Tempranillo.

As observed in the Figure 2, TA and °Brix followed the expected trend: TA decreased along the ripening process, while sugar content increased. °Brix/TA ratio increased accordingly. These values were stabilized after week 5, indicating the proximity of the end of ripeness, and grapes were considered mature and harvested in week 6. Similar trends were observed in Garnacha and Cabernet-Sauvignon that also reached the maturity in week 6. Mencia Secano, Mencia Regadio, Rufete, and Juan Garcia gapes, matured at a faster rate, and were considered ripened and harvested in week 5.

In order to study the changes in over-ripened grapes, some bunches were kept in the plant during 2 more weeks. During these 2 extra weeks, TA values were almost constant, whereas °Brix raised drastically in Cabernet and Garnacha grapes, indicating that optimal ripening was not attained until weeks 7 or 8.

TPI values increased sharply just after the *véraison*. The following weeks, TPI varied irregularly. These results evidenced the difficulty to assess the phenolic maturity using traditional TPI measurements in pulps. It is therefore important for wine producers to develop new methods to define the peak of ripeness and in particular of the phenolic ripeness (which nowadays is difficult to assess).

### Electrochemical characterization of the NP-based electrode. electrocatalytic activity toward phenols

**Figure 3:**
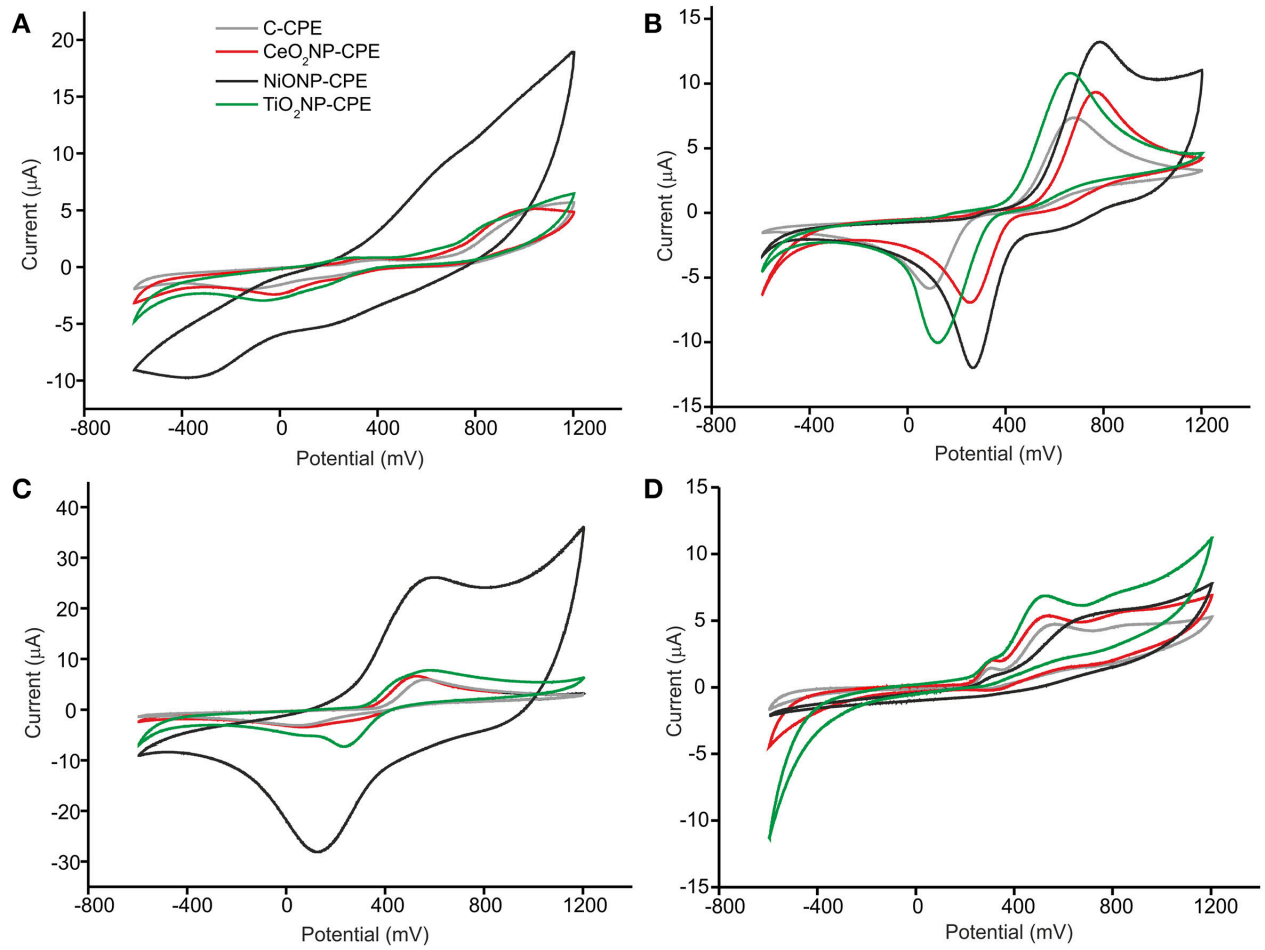
CV of **(A)** vanillic acid, **(B)** catechol, **(C)** caffeic acid, and **(D)** pyrogaloll at C-CPE, CeO_2_NP-CPE, NiONP-CPE, and TiO_2_NP-CPE electrodes. Concentration of 10^−4^M. Scan rate 100 mV·s^−1^.

The objective of this work was to develop a multisensory system based on metal oxide nanoparticles to monitor the phenolic ripening. As a first task, the response of the MONP-CPE electrodes toward four phenols present in musts (vanillic acid, catechol, caffeic acid, and pyrogallol 10^−4^ M), was evaluated (Figure 3). The response of the C-CPE electrode is also shown for comparison purposes.

The responses on MONP-CPEs were consistent with those already reported in bare CPE electrodes (Kilmartin et al., 2001), but the presence of MONPs caused shifts in the position of the peaks to lower potentials and/or increases in the intensity of the peaks. This effect could be attributed to the excellent electrocatalytic activity of MONPs due to the mixed valence state found at the nanoparticle surface (Medina-Plaza et al., 2015b). Depending on the nature of the MONPs used as modifiers, the responses toward a certain compound was more or less enhanced. For instance, the intensity of the redox peaks produced by the oxidation/reduction observed in vanillic acid and catechol at the graphite electrode surface increased drastically in the presence of NiONPs. The electrocatalytic effect was not so remarkable at TiO_2_NP-CPE or CeO_2_NP-CPEs electrodes. It is also worth noting that the electrocatalytic effect was more intense in the mono-phenol (vanillic acid) or di-phenols (catechol and caffeic acid) than in the tri-phenol (pyrogallol).

**Figure 4:**
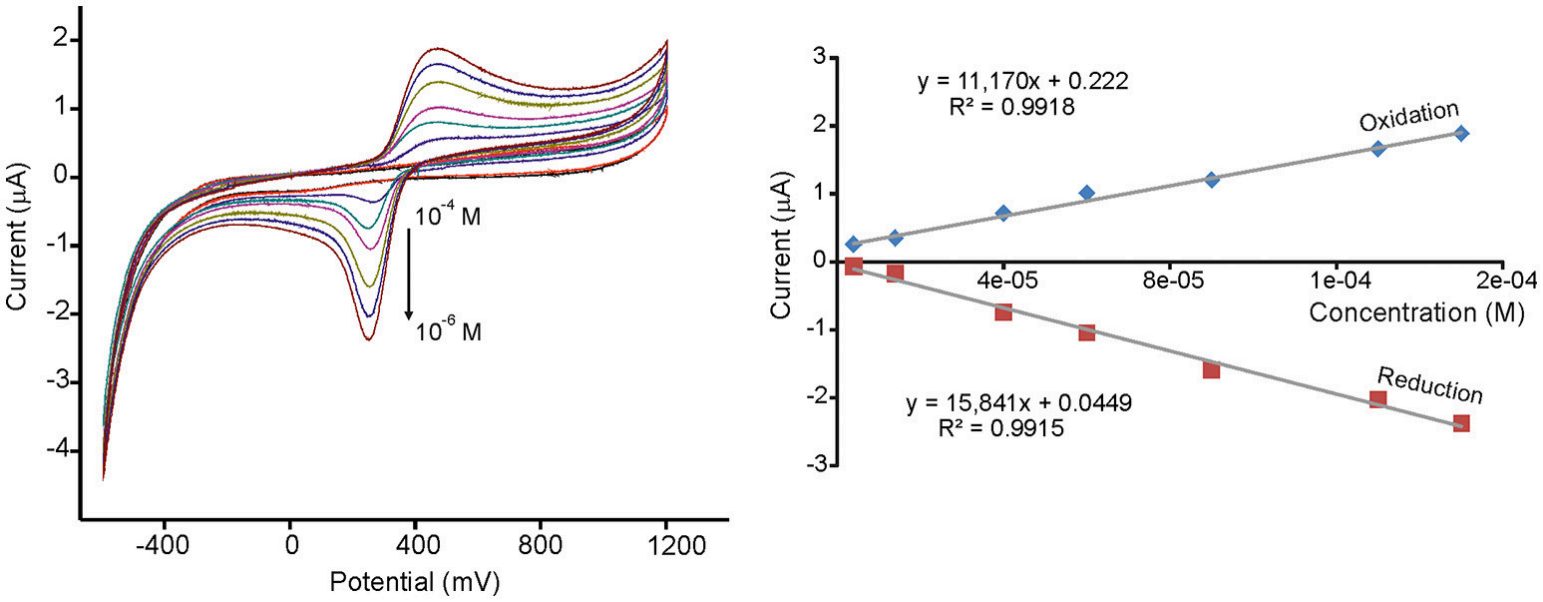
CVs and calibration curves of TiO_2_NP-CPE immersed in increasing concentrations of caffeic acid (from 4.0·10^−6^ M to 1.5·10^−4^ M). Scan rate 0.1 V·s-1.

Limits of detection (LD) were calculated from the slope of the curves of the redox peaks registered at concentrations ranging from 10^−4^ to 10^−6^ M, using 3 *SD*/m method, where *SD* is the standard deviation of the current density. LDs as low as 10^−8^ M could be attained, which are much lower than those typically found in carbon electrodes (Kilmartin et al., 2001). The results are illustrated in Figure 4 for TiO_2_NP-CPE immersed in caffeic acid. Reproducibility was also evaluated by measuring repeatedly the standard solutions and Variation coefficients (CV) lower than 7% were found in all cases. From the above results it can be concluded that modification with metal oxide nanoparticles drastically increases the sensibility of voltammetric electrodes toward phenols present in musts.

As stated before, each sensor showed a characteristic response which is linked to the electrocatalytic properties of the type of nanoparticle introduced in the carbon paste. This cross-selectivity can be used to construct a multisensor system coupled to a pattern recognition software able to provide a pattern or “digital fingerprint” for each sample studied.

**Figure 5:**
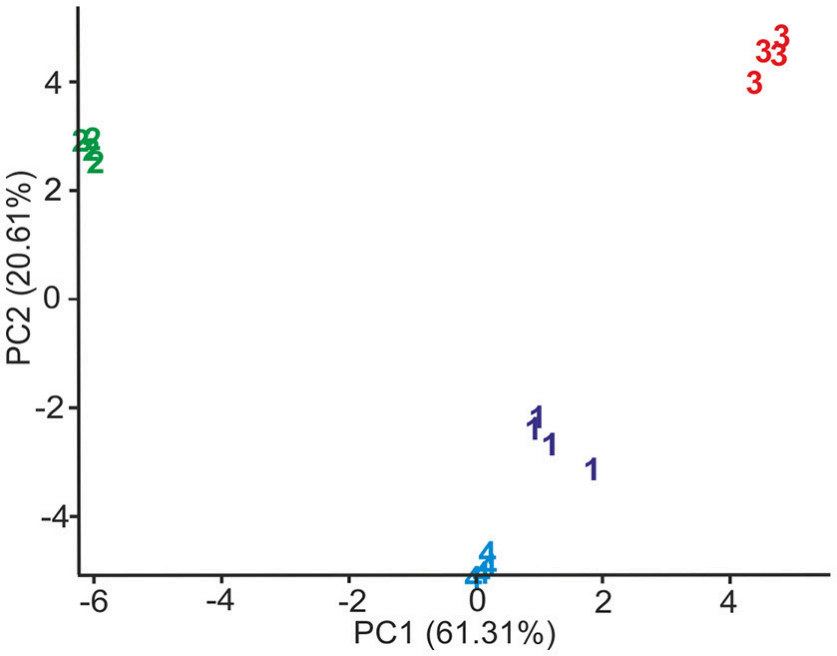
Scores plot of the first two principal components of the PCA model calculated from voltammograms registered when electrodes were immersed in 1: Catechol, 2: Pyrogallol, 3: Vanillic acid and 4: Caffeic acid.

Figure 5 shows the Principal Component Analysis (PCA) scores plot obtained from the responses of the array to 10^−4^ M solutions of phenols. The PCA showed four distinct clusters in the plot of the two first principal components (PC1 61.31% and PC2 20.61%). The position of the clusters was related to the number of hydroxyl groups in the molecule. The monophenol (vanillic acid) was located at positive PC1 values, diphenols (catechol and caffeic acid) in the central part of the diagram and the triphenol (pyrogallol) on the left side corresponding to positive PC2 values.

### Monitoring the ripening of grapes with an array of MONP-based electrodes

The capacity of the electronic tongue to monitor the phenolic maturity of grapes was evaluated by analyzing juices obtained from grapes collected from *véraison*, in a weekly basis, during 7–8 weeks. Due to the complexity of the samples and in order to improve the repeatability, musts were diluted 1:2 with deionized water. Coefficients of variation lower than 7% were obtained in all cases.

**Figure 6:**
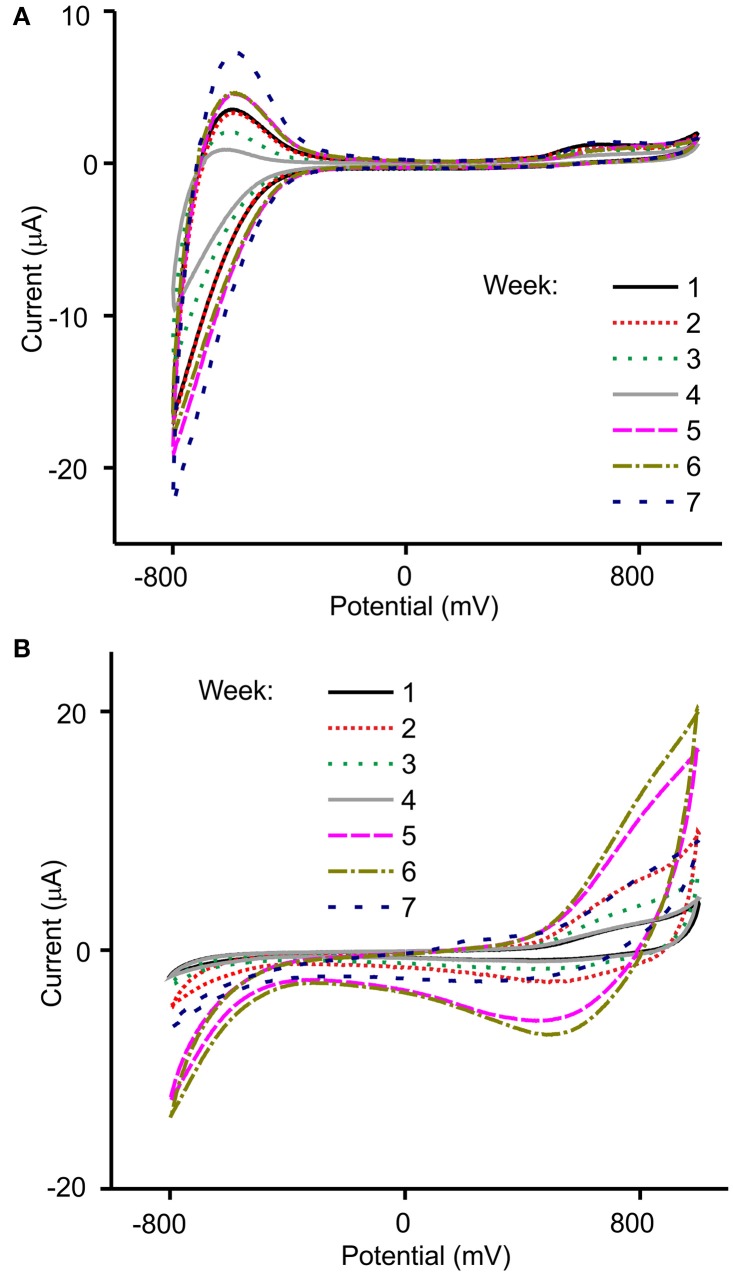
Voltammetric responses of **(A)** TiO_2_NP-CPE and **(B)** NiONP-CPE electrodes toward Garnacha grapes collected weekly after *véraison*.

Figure 6 illustrates the responses obtained using the NiONP-CPE and TiO_2_NP-CPE sensors immersed in musts obtained from grapes of the variety Garnacha collected during 7 weeks after *véraison*.

As illustrated in the figure, voltammograms registered in must showed peaks in the 0.6–0.8 V region, produced by components with redox activity (mainly phenols). Other components present in musts also influenced the overall response. For instance, the peaks observed at negative potentials were affected by the acidity of the samples. The array of sensors showed an interesting cross-selectivity that was due to the different catalytic properties of the MONP selected. For instance, NiONP-CPEs were highly sensitive to phenols, whereas TiO_2_NP-CPEs were sensitive to the acidity.

The chemical changes occurring during the ripening process could be followed using the MONP-based sensors. First, the intensity of the peaks increased, then intensity decreased and finally increased according with the tendency shown in Figure 2 where TPI values varied similarly. The irregular increase stopped when optimal ripeness was attained. Simultaneously, a decrease in the total acidity corresponded with a progressive decrease in the intensity of the peaks at negative potentials. As already stated in the introduction, multivariate methods have demonstrated to be a good strategy for the analysis of wines. It can be expected that they can also be useful to monitor the ripeness of grapes.

**Figure 7:**
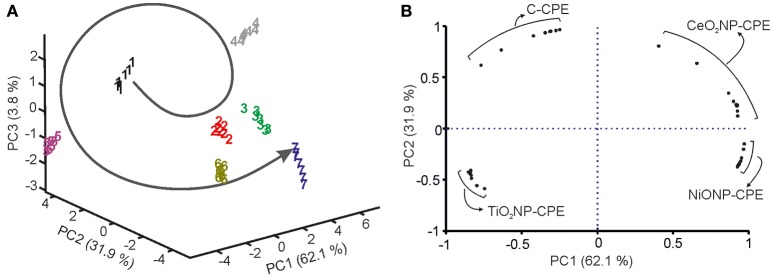
**(A)** 3D Score plot, **(B)** Loading plot of PCA performed from grape juices from the variety Juan Garcia harvested weekly from *véraison* to over-ripening (1: First week, 2: Second week, etc. 7: Over-ripening week).

When PCA was carried out using the data obtained from the electronic tongue, it was observed that clusters corresponding to grapes collected in the first 4 weeks after *véraison*, were positioned in a counterclockwise organization “drawing” a circle (Figure 7A). In sampling number 5, the circle was closed. Surprisingly, in next samplings the positions were repeated and clusters corresponding to next specimens were situated continuing the counterclockwise trend following a “screw” structure. According to PCA, grapes could be considered matured when the clusters reached the original positions. Similar results have been obtained for all the grape varieties analyzed in this work. Similar results have also been observed in previous works where ETs have been used to monitor maturity of grapes (Medina-Plaza et al., 2016)

Figure 7B show the contribution of the variables in a loading plot. The loadings of each sensor (ten variables per sensor) spread along the four quadrants. This graph shows the high cross-selectivity achieved by the array of sensors.

Table 1 shows the statistical parameters obtained from the PLS-1 regression models (leave one out validation) correlating the output of the array of sensors with the chemical parameters (TPI, °Brix, TA). PLS-1 builds regression models to establish a linear relationship between the set of predictors (X-variable: Voltammetric signals obtained from the array of sensors) and the set of responses (Y-variable: Chemical parameters obtained with traditional methods). PLS-1 finds the latent variables (indicated as number of components) in X that will best predict the latent variables in Y.

**Table 1:** Results of the PLS-1 regression models correlating the chemical parameters and the signals of the sensors.

**Grape**	**Parameter**	**$R_{\text{C}}^{2}$(a)**	**RMSE_C_(b)**	**$R_{\text{P}}^{2}$(C)**	**RMSE_P_(d)**	**Number of components**
Cabernet	°Brix	0.87	0.86	0.85	0.95	**3**
	TA (g/L)	0.87	0.81	0.81	1.02	7
	TPI	0.86	2.13	0.83	2.42	**2**
Garnacha	°Brix	0.92	0.91	0.82	1.40	6
	TA (g/L)	0.85	1.24	0.73	1.72	6
	TPI	0.86	2.25	0.77	3.01	**3**
Juan Garcia	°Brix	0.86	0.79	0.79	1.00	5
	TA (g/L)	0.80	1.08	0.70	1.39	6
	TPI	0.84	1.54	0.79	1.84	**4**
Mencia Regadio	°Brix	0.97	0.22	0.96	0.26	5
	TA (g/L)	0.89	0.69	0.86	0.77	5
	TPI	0.88	1.08	0.83	1.33	5
Mencia Secano	°Brix	0.86	0.58	0.79	0.74	6
	TA (g/L)	0.81	0.84	0.71	1.06	6
	TPI	0.86	1.57	0.82	1.79	**3**
Prieto Picudo	°Brix	0.87	0.73	0.63	1.25	7
	TA (g/L)	0.88	0.93	0.70	1.52	7
	TPI	0.83	1.33	0.81	1.43	**3**
Rufete	°Brix	0.85	0.49	0.83	0.53	**3**
	TA (g/L)	0.82	0.91	0.76	1.09	5
	TPI	0.91	1.51	0.90	1.64	**3**
Tempranillo	°Brix	0.81	0.89	0.76	1.01	6
	TA (g/L)	0.91	0.56	0.76	0.95	6
	TPI	0.87	2.05	0.75	2.94	**4**

*Models were established for each variety of grape considered separately*.

*(a) Squared correlation coefficient in calibration; (b) Root mean square error of calibration; (c) Squared correlation coefficient in prediction; (d) Root mean square error of prediction*.

In all varieties of grapes, good correlations were found with the Total Polyphenol Index. Using a low number of components, both the calibration and the prediction showed Squared Correlation Coefficients in calibration (R^2^C) and in prediction (R^2^V) of ca. 0.8. Additionally, low Root Mean Square Error of calibration (RMSEC) and of prediction (RMSEp) were accomplished. These results confirm that the ET can be used to monitor the phenolic maturity of grapes. The °Brix and Total Acidity which are the parameters commonly used to evaluate the maturity of grapes, could also be predicted using the PLS-1 models but with lower correlation coefficients in prediction (~0.7) and the models need a higher number of components. In spite of the lower correlation, the models are good enough to confirm that the electrochemical responses also reflected the acidity and the sugar concentration. This indicates that it is possible to obtain simultaneous information of the three main parameters used to monitor the maturity of grapes.

## Conclusions

The advantages of metal oxide nanoparticles as sensing materials for the detection of phenols has been evidenced. They rely on the excellent electrocatalytic properties of the metal oxide nanoparticles, that improved the sensitivity and limits of detection toward phenols with respect to unmodified sensors. Using these exceptional sensing properties, and the cross-selectivity shown by different MONPs, an electronic tongue based on metal oxide nanoparticles has been developed. The chemical changes occurring during ripening could be followed using the MONP-based multisensor system. The sensors based on different MONP show an excellent degree of cross-selectivity. Using PLS-1, correlations have been established between the signal of the multisensor system and the Total Polyphenol Index TPI, the °Brix, and the Total Acidity.

According to this, simultaneous analysis of three important markers of maturity can be achieved using a multisensory system based on MONPs. This means that the ET can be a useful tool to establish with a better accuracy the optimal harvesting date in a single experiment. This method can be used as a supplementary tool to classical analytical techniques.

The results shown here demonstrate the excellent opportunities offered by metal oxide nanoparticles as sensing materials in electronic tongues.

## Author contributions

MR-M: conceived the experiments an wrote the paper; CG-H, CM-P, CG-C, and YB: carried out the experiments; JF-E and EB-T: provided de grape samples and analyzed the grapes by chemical methods; MR-P and FM-P: carried out the chemometric analysis.

### Conflict of interest statement

The authors declare that the research was conducted in the absence of any commercial or financial relationships that could be construed as a potential conflict of interest.
