# Supplementary material for: Monitoring the Phenolic Ripening of Red Grapes Using a Multisensor System Based on Metal-Oxide Nanoparticles
Source: Front Chem. 2018 Apr 24;6:131. doi: 10.3389/fchem.2018.00131 (PMC5928143; doi:10.3389/fchem.2018.00131)
Supplement: Supplementary file 1 [file Table_1.docx]

**Supplementary Table.** Chemical analysis of grape samples collected during 2014 vintage.

| **Variety** | **Sampling**  **week** | **Sample**  **name** | **ºBrix** | **TA (g/L)** | **ºBrix/TA** | **IPT** |
| --- | --- | --- | --- | --- | --- | --- |
| Cabernet | 1 | C1 | 17.50 | 13.11 | 1.33 | 3 |
|  | 2 | C2 | 20.00 | 10.86 | 1.84 | 20 |
|  | 3 | C3 | 21.80 | 9.53 | 2.28 | 17 |
|  | 4 | C4 | 21.80 | 8.10 | 2.69 | 17 |
|  | 5 | C5 | 23.40 | 6.39 | 3.66 | 19 |
|  | 6 (*) | C6 | 22.40 | 6.30 | 3.55 | 14 |
|  | 7 | C7 | 23.10 | 7.03 | 3.28 | 18 |
|  | 8 | C8 | 26.20 | 7.19 | 3.64 | 24 |
| Garnacha | 1 | G1 | 15.60 | 16.05 | 0.97 | 3 |
|  | 2 | G2 | 16.30 | 13.93 | 1.17 | 25 |
|  | 3 | G3 | 18.10 | 11.12 | 1.62 | 16 |
|  | 4 | G4 | 19.20 | 8.39 | 2.28 | 16 |
|  | 5 | G5 | 20.30 | 7.20 | 2.81 | 18 |
|  | 6 (*) | G6 | 20.20 | 8.19 | 2.46 | 17 |
|  | 7 | G7 | 21.60 | 8.47 | 2.55 | 18 |
|  | 8 | G8 | 26.80 | 6.03 | 4.44 | 23 |
| Juan Garcia | 1 | JG1 | 16.20 | 12.43 | 1.30 | 6 |
|  | 2 | JG2 | 18.50 | 9.76 | 1.89 | 18 |
|  | 3 | JG3 | 20.30 | 7.23 | 2.80 | 13 |
|  | 4 | JG4 | 23.10 | 6.10 | 3.78 | 14 |
|  | 5 (*) | JG5 | 21.10 | 5.43 | 3.88 | 11 |
|  | 6 | JG6 | 21.40 | 5.64 | 3.79 | 17 |
|  | 7 | JG7 | 21.80 | 6.07 | 3.59 | 17 |
| Mencia Regadio | 1 | MR1 | 18.60 | 9.66 | 1.92 | 6 |
|  | 2 | MR2 | 20.10 | 8.70 | 2.31 | 17 |
|  | 3 | MR3 | 21.80 | 5.92 | 3.68 | 13 |
|  | 4 | MR4 | 22.00 | 7.35 | 2.99 | 12 |
|  | 5 (*) | MR5 | 22.40 | 4.71 | 4.75 | 14 |
|  | 6 | MR6 | 21.70 | 4.22 | 5.14 | 14 |
|  | 7 | MR7 | 22.50 | 4.24 | 5.30 | 12 |
| Mencia Secano | 1 | MS1 | 18.00 | 10.30 | 1.74 | 6 |
|  | 2 | MS2 | 20.50 | 7.37 | 2.78 | 20 |
|  | 3 | MS3 | 21.90 | 6.79 | 3.22 | 17 |
|  | 4 | MS4 | 20.80 | 5.77 | 3.60 | 15 |
|  | 5 (*) | MS5 | 23.00 | 4.91 | 4.68 | 14 |
|  | 6 | MS6 | 22.90 | 4.61 | 4.96 | 15 |
|  | 7 | MS7 | 21.30 | 4.56 | 4.67 | 18 |
| Prieto Picudo | 1 | PP1 | 17.30 | 13.92 | 1.24 | 3 |
|  | 2 | PP2 | 19.40 | 11.25 | 1.72 | 11 |
|  | 3 | PP3 | 22.20 | 9.07 | 2.44 | 9 |
|  | 4 | PP4 | 22.00 | 5.90 | 3.72 | 14 |
|  | 5 (*) | PP5 | 23.00 | 6.91 | 3.32 | 8 |
|  | 6 | PP6 | 22.90 | 6.62 | 3.45 | 10 |
|  | 7 | PP7 | 22.80 | 7.03 | 3.24 | 12 |
| Rufete | 1 | R1 | 18.10 | 11.91 | 1.51 | 6 |
|  | 2 | R2 | 18.70 | 9.13 | 2.04 | 22 |
|  | 3 | R3 | 20.40 | 7.81 | 2.61 | 14 |
|  | 4 | R4 | 22.20 | 5.34 | 4.15 | 19 |
|  | 5 (*) | R5 | 20.00 | 5.78 | 3.46 | 11 |
|  | 6 | R6 | 20.60 | 5.74 | 3.58 | 18 |
|  | 7 | R7 | 20.80 | 7.58 | 2.74 | 17 |
| Tempranillo | 1 | T1 | 17.90 | 11.24 | 1.59 | 3 |
|  | 2 | T2 | 19.00 | 9.83 | 1.93 | 20 |
|  | 3 | T3 | 20.70 | 8.01 | 2.58 | 16 |
|  | 4 | T4 | 22.10 | 6.29 | 3.51 | 17 |
|  | 5 | T5 | 22.40 | 5.75 | 3.89 | 19 |
|  | 6 (*) | T6 | 22.80 | 6.53 | 3.49 | 24 |
|  | 7 | T7 | 23.40 | 5.56 | 4.20 | 18 |
|  | 8 | T8 | 24.20 | 7.03 | 3.44 | 19 |

(*) Indicates the optimal date of harvest decided by the oenoligist, according to traditional methods.
